# ESA-YOLOv5m: a lightweight spatial and improved attention-driven detection for brain tumor MRI analysis

**DOI:** 10.3389/fmed.2025.1733180

**Published:** 2025-12-19

**Authors:** Maram Fahaad Almufareh, Noshina Tariq, Mamoona Humayun, Haya Aldossary, Meshal Alharbi

**Affiliations:** 1 Department of Information Systems, College of Computer and Information Sciences, Jouf University, Sakaka, Saudi Arabia; 2 Department of Artificial Intelligence and Data Science, National University of Computer and Emerging Sciences, Islamabad, Pakistan; 3 School of Computing, Engineering and the Built Environment, University of Roehampton, London, United Kingdom; 4 Computer Science Department, College of Science and Humanities, Imam Abdulrahman Bin Faisal University, Al Jubail, Saudi Arabia; 5 Department of Computer Science, College of Computer Engineering and Sciences, Prince Sattam Bin Abdulaziz University, Al-Kharj, Saudi Arabia

**Keywords:** YOLOv5m, Enhanced Spatial Attention (ESA), brain tumor detection, medical imaging, deep learning, precision and recall, mAP, Figshare MRI dataset

## Abstract

**Introduction:**

The early and accurate detection of brain tumors is vital for improving patient outcomes, enabling timely clinical interventions, and reducing diagnostic uncertainty. Despite advances in deep learning, conventional Convolutional Neural Network (CNN)-based models often struggle with small or low-contrast tumors. They also remain computationally demanding for real-time clinical deployment.

**Methods:**

This study presents an Enhanced Spatial Attention (ESA)-integrated You Only Look Once v5 medium (YOLOv5m) architecture, a lightweight and efficient framework for brain tumor detection in MRI scans. The ESA module, positioned after the Spatial Pyramid Pooling-Fast (SPPF) layer, enhances feature discrimination by emphasizing diagnostically relevant regions while suppressing background noise, thereby improving localization accuracy without increasing computational complexity. Experiments were conducted on the Figshare brain tumor MRI dataset containing three tumor classes: glioma, meningioma, and pituitary.

**Results:**

ESA-YOLOv5m achieved a Precision of 90%, Recall of 90%, and mean Average Precision (mAP)@0.5 of 91%, surpassing the baseline YOLOv5m by approximately 11%–12%. An ablation study further confirmed that placing the ESA module after the SPPF layer yields the highest performance (mAP@0.5 = 0.91), while earlier integration produced marginally lower results. Classwise analyses demonstrated consistent gains (mAP range 0.87–0.98), and fivefold cross-validation showed stable performance (mAP@0.5 = 0.910 ± 0.006). Efficiency tests revealed negligible overhead, with less than a 4.3% increase in parameters and an average latency below 10 ms per image.

**Discussion:**

Overall, the results validate that integrating a lightweight spatial attention mechanism significantly enhances tumor localization and model generalization while preserving real-time inference. The proposed ESA-YOLOv5m framework provides a reliable and scalable solution for automated brain tumor detection, suitable for clinical decision-support systems and edge healthcare applications.

## 1 Introduction

The integration of artificial intelligence (AI) and Deep Learning (DL) into medical imaging has transformed disease diagnosis, enabling rapid and accurate clinical assessment (1). Among the various applications, brain tumor detection has gained particular importance, as early and reliable diagnosis directly influences patient survival and treatment outcomes. Brain tumors, whether benign or malignant, remain one of the most critical neurological conditions, often requiring timely surgical or therapeutic intervention (2). Magnetic Resonance Imaging (MRI) continues to be the preferred non-invasive modality for brain tumor analysis. It provides high soft-tissue contrast and detailed anatomical representation (3). However, manual interpretation of MRI scans is labor-intensive and subjective. It is also prone to inter-observer variability, especially in cases involving small, irregular, or low-contrast lesions (4). These limitations have motivated the development of automated and intelligent diagnostic system. Such systems are capable of assisting radiologists in precise tumor localization and classification.

Brain tumors are a serious health risk to the human body, and when they are not identified in time, they cause irreparable neurological conditions or even death. Early and accurate diagnosis is thus not only preferable but also required to enhance patients' survival rates and to provide them with timely intervention, which is less invasive. This clinical urgency supports the fact that powerful Computer-Aided Diagnosis (CAD) systems are necessary to help radiologists quickly and effectively detect tumors. Early CAD frameworks relied on handcrafted features and classical machine learning algorithms, which were insufficient to capture the heterogeneous appearance of brain tumors across different patients and imaging conditions (5, 6). DL, particularly CNNs, has since become the dominant approach in medical image analysis due to its ability to learn hierarchical feature representations directly from raw data (7). In this paradigm, object detection architectures such as You Only Look Once (YOLO) have shown exceptional efficiency for real-time medical image interpretation because of their end-to-end design and low inference latency (8, 9).

Nevertheless, standard CNN-based detectors encounter persistent challenges when applied to medical imaging (10). Tumors vary significantly in size, texture, and intensity, and are often embedded within complex anatomical structures (11). These characteristics make small tumor regions difficult to detect, frequently leading to false negatives (12, 13). Additionally, existing high-performance CNNs are computationally demanding, limiting their adoption in clinical environments or on edge devices where real-time processing is essential. To overcome these limitations, recent studies have explored hybrid and attention-enhanced frameworks (14), yet many of these remain computationally heavy and lack consistent generalization across tumor types.

To address these challenges, this study proposes an Enhanced Spatial Attention (ESA)-integrated YOLOv5m model that enhances spatial focus during tumor localization. The ESA module is inserted after the Spatial Pyramid Pooling-Fast (SPPF) layer, enabling the network to prioritize diagnostically important regions while suppressing irrelevant background. This design strengthens the model's sensitivity to small and low-contrast tumors without introducing significant computational overhead. The lightweight structure of ESA-YOLOv5m ensures that it remains deployable in real-time and resource-constrained healthcare settings. In Clinical practice, supplementary spatial attention aids physicians by automatically highlighting critical tumor margins and suppressing distracting structures in phantoms. This not only lessens time spent on delineating lesions, but also alleviates inter-observer variability in contouring difficult or low contrast lesions. This enables radiologists to concentrate on the areas the algorithms identify and subsequently verify, thus reducing the time taken to report while also reducing uncertainty in diagnosis during the screening or follow-up examinations. A comprehensive series of experiments was performed on the Figshare brain tumor MRI dataset, comprising three tumor types: glioma, meningioma, and pituitary. Comparative evaluations with the baseline YOLOv5m demonstrated clear improvements in Precision, Recall, and mean Average Precision at an IoU threshold of 0.5 [mean Average Precision (mAP)@0.5]. Furthermore, an ablation study confirmed that the optimal performance was achieved when ESA was positioned after the SPPF layer, leading to the highest detection accuracy (mAP@0.5 = 0.91) while maintaining low latency. Class-wise analysis indicated consistent gains across all tumor categories, and five-fold cross-validation established the model's stability and reproducibility. The main contributions of this study are summarized as follows:

1. The backbone of YOLOv5m is modified via the Enhanced Spatial Attention (ESA) module to increase localization precision and detection of small or low-contrast tumors in MRI images.
2. Extensive ablation experiments show that the best generalization and accuracy at the lowest extra cost of computation occurs with ESA placement after the SPPF layer.
3. ESA-YOLOv5m maintains the ability to perform real-time inferences and has higher Precision, Recall, and mAP@0.5 values than the baseline YOLOv5m.
4. The robustness and stability of the model across diverse tumor classes are shown through comprehensive evaluations, including class-wise and five-fold cross-validation analyses.
5. ESA-YOLOv5m's ultralightweight design and high efficiency allow it to be used in direct clinical applications and deployed on edge computing medical devices.

### 1.1 Paper organization

The remainder of this paper is structured as follows: Section 2 sheds light on related work, Section 4 presents the methodology, including data preprocessing, model architecture, and training strategy. Section 5 describes the experimental setup and hyperparameters. Section 6 provides the results and detailed discussion of the model's performance. Finally, Section 7 concludes the paper and discusses future research directions.

## 2 Related work

Automated brain tumor detection on MRI has attracted growing attention in recent years, driven by the rapid progress of deep learning and computational imaging. Early computer-aided diagnosis (CAD) approaches primarily relied on handcrafted features and classical machine learning models such as support vector machines (SVMs) and random forests. Although these models offered interpretability, they struggled to generalize across datasets with varying imaging parameters and tumor morphologies. For instance, Hussain et al. (15) employed feature-based classifiers for tumor recognition but reported limited robustness under different scanner settings. Later, Muhammad et al. (16) conducted a comprehensive survey confirming that convolutional neural networks (CNNs) outperform traditional techniques in most MRI-based tumor classification tasks. Similarly, Yildirim et al. (17) achieved per-class precision between 0.82 and 0.93 using a ResNet-based hybrid CNN model for glioma, meningioma, and pituitary tumor classification, highlighting the advantages of deep hierarchical features over manually engineered descriptors.

Despite these improvements, CNN-based models often face challenges in accurately identifying small or low-contrast tumors, which frequently leads to false negatives (7). Moreover, large network architectures are computationally intensive, restricting their use in real-time or resource-constrained environments such as clinical workstations and edge healthcare systems. To overcome these limitations, attention and transformer-based mechanisms have gained popularity for enhancing spatial awareness and model interpretability (13).

Recent studies have explored lightweight attention-driven architectures to refine feature representation without incurring excessive computational costs. Hekmat et al. (18) introduced an Attention-Fused MobileNet-LSTM framework that combines convolutional and recurrent layers to capture both spatial and temporal dependencies in MRI sequences, achieving 98.66% accuracy. Although their model demonstrated strong interpretability through Grad-CAM visualization, the LSTM component increased training complexity and reduced transparency in feature extraction. Similarly, Dutta et al. (19) proposed ARM-Net, an attention-guided residual multiscale CNN that emphasized class-specific tumor features, improving multi-class accuracy on the Figshare dataset but at the expense of greater computational demand.

Vision transformer (ViT) models have also emerged as powerful alternatives by modeling long-range spatial dependencies. Poornam and Angelina (20) proposed the VITALT model, which combines self-attention with linear transformations to capture both local and global MRI features. Although effective, transformer-based methods are computationally expensive and may not be suitable for real-time deployment. Complementary approaches have incorporated hybrid attention mechanisms; for instance, Saeed et al. (21) developed GGLA-NeXtE2NET, a dual-branch ensemble integrating gated global-local attention with EfficientNet and ConvNeXt backbones, achieving over 99% accuracy in multi-class classification. While such models capture multiscale contextual information, their heavy architecture and reliance on GAN-based augmentation (ESRGAN) limit deployment feasibility.

In parallel, studies employing object detection frameworks such as the YOLO family have demonstrated promising results in localizing tumors directly on MRI images. YOLO-based architectures have been adapted for real-time detection of glioma, meningioma, and pituitary lesions, offering an optimal trade-off between accuracy and inference speed (9). As illustrated in Rastogi et al. (2), the combination of transfer learning and fine-tuning techniques improved precision in detection even in the absence of large datasets. This is very pertinent as new hybrid architectures with certain secure and privacy-protected modules (14) emphasize the increasing demand for clinically relevant and low-computation systems in distributed medical settings.

Table 1 summarizes representative studies on brain tumor detection and classification, outlining their core methodologies, attention mechanisms, performance metrics, and limitations. Although recent models have achieved high accuracy, most remain computationally demanding or lack robustness when detecting small and irregular tumor regions. Existing research demonstrates the rapid evolution from classical handcrafted-feature methods to deep attention-based and transformer-driven architectures for brain tumor analysis. While transformer and ensemble approaches (20, 21) achieve excellent accuracy, they typically require high computational power and large annotated datasets. Hybrid CNN-LSTM and multiscale networks (18, 19) improve interpretability but remain unsuitable for real-time inference due to their complexity. Similarly, YOLO-based detectors (9) achieve good speed-accuracy trade-offs but still struggle with small or low-contrast tumors. The proposed ESA-YOLOv5m model integrates Enhanced Spatial Attention (ESA) mechanisms into the architecture of YOLOv5m. This design maintains model efficiency while also increasing the model's sensitivity to small and diffuse regions of the tumor. Strong ablation studies have shown that the ideal position for the ESA block is after the SPPF layer. This achieves the best balance of the model accuracy, interpretability, and real-time applicability. As a result, ESA-YOLOv5m is a robust, scalable model that applies to practical conditions, effectively addressing the performance-efficiency gap identified in the current state-of-the-art approaches.

**Table 1:** Comparison of recent brain tumor detection and classification studies (2019–2025).

**References**	**Architecture**	**Attention/mechanism**	**Dataset**	**Accuracy/mAP**	**Key limitations**
Hussain et al. (15)	Feature-based ML (SVM, RF)	None	Private MRI	89.4% (accuracy)	Limited generalization and noise sensitivity
Yildirim et al. (17)	ResNet-based hybrid CNN	None	Figshare MRI	82%–93% (precision)	Weak detection of small tumors
Hekmat et al. (18)	MobileNet-LSTM hybrid	Attention fusion + Grad-CAM	Public MRI	98.66% (accuracy)	High complexity, limited generalization
Dutta et al. (19)	ARM-Net (residual multiscale CNN)	Channel-spatial attention	Figshare MRI	91.5% (accuracy)	Heavy model, lacks real-time feasibility
Poornam and Angelina (20)	Vision transformer (VITALT)	Self-attention (transformer blocks)	4-class MRI	96.2% (accuracy)	Computationally intensive, not real-time
Saeed et al. (21)	GGLA-NeXtE2NET (ensemble)	Gated global-local attention	4-class MRI	99.0% (accuracy)	High memory demand, complex training
Rastogi et al. (2)	Fine-tuned CNN (transfer learning)	None	Kaggle MRI	97.3% (accuracy)	Limited to image-level classification
Saranya and Praveena (9)	YOLOv5/YOLOv7 detectors	None	MRI (3-class)	mAP@0.5 ≈ 0.90	Recall drops for diffuse tumors
This study	YOLOv5m + ESA module	Enhanced Spatial Attention (ESA)	Figshare MRI	mAP@0.5 = 0.91, Precision = 0.90, Recall = 0.90	Lightweight, real-time, effective on small lesions

More recent developments in the YOLO family, including v3, v4, v5, v6, v7, and v8, have brought about the use of anchor-free detection heads and decoupled classification-regression branches as well as enhanced feature-pyramid aggregation. A number of medical-imaging works have used these variations on domain-specific tasks, including the tumor grading task (22–24), the task of occupational safety (22, 25), and the task of human-falls detection (26, 27). Nonetheless, the methods place significant emphasis on accuracy and minimal focus on interpretability and computation efficiency, which are important in clinical translation. Our ESA-YOLOv5m helps overcome these flaws with a lightweight attention-based on the improvement of the spatial focus without raising the inference latency. As discussed earlier, previous research illustrates architectural evolution. However, there is a gap in primary research that relates to lightweight spatial-attention mechanisms designed for medical YOLO models. Most existing transformer and hybrid models focus on accuracy at the expense of practical deployment, and earlier YOLO versions forgo detailed spatial refinement. The ESA-YOLOv5m aims at closing these gaps by introducing an attention block that is interpretable and resource-efficient, and that improves tumor localization in real-time.

## 3 Proposed model architecture

**Figure 1:**
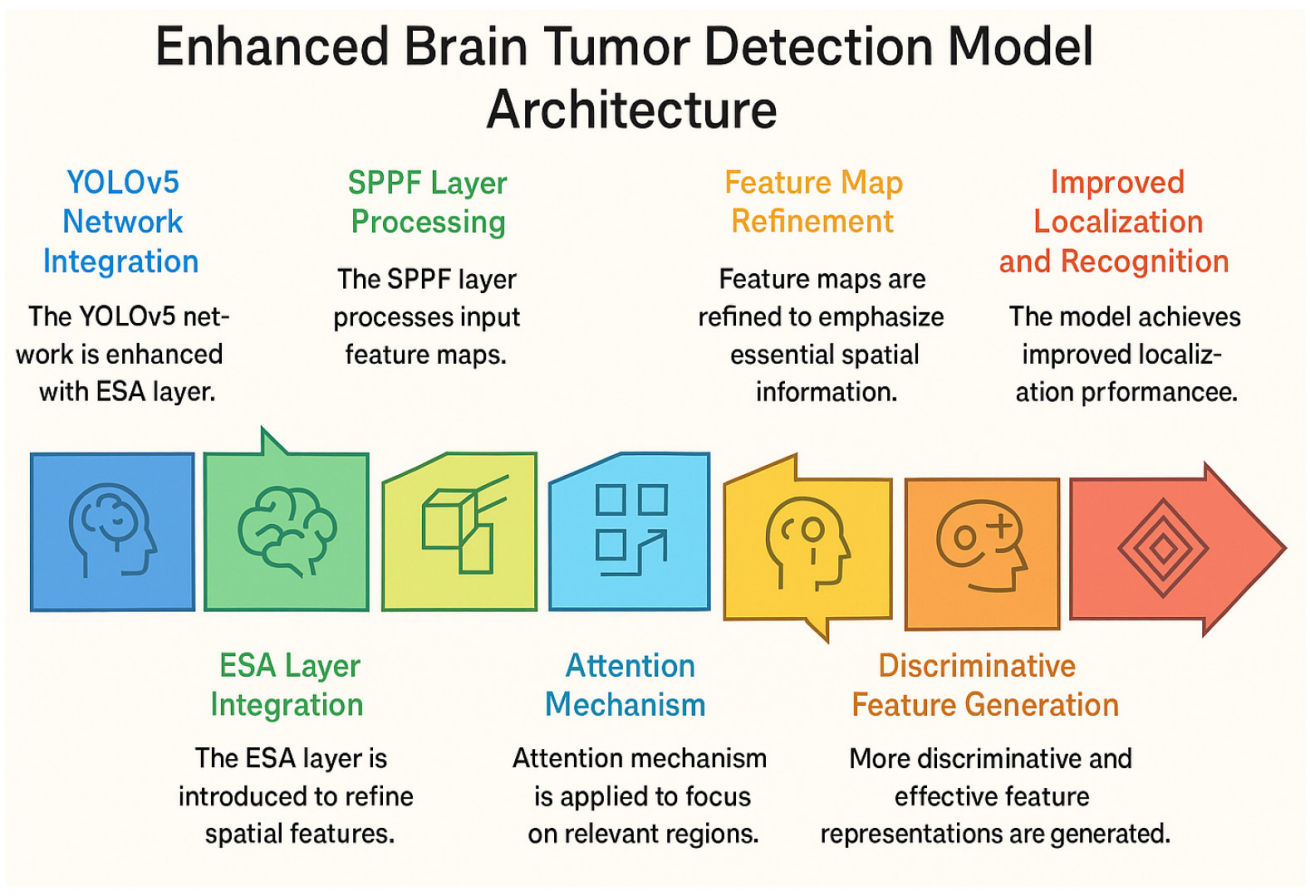
ESA-YOLOv5m architecture with the ESA block integrated to enhance spatial feature refinement for brain tumor localization.

The proposed model aims to detect brain tumors from MRI slices with greater accuracy and efficiency than existing methods, with the integration of Enhanced Spatial Attention to the current YOLOv5m configuration, as shown in Figure 1. This architecture aims to fast extract “meta” features while performing “spatial” attention refinement to maximize attention “focus” (localization) and minimize “time” (latency) to soothingly generalize (low) MRI dataset variability (across). While the latest detectors YOLOv8 and YOLOv9 boast near parity improvements in accuracy, for the purpose of this study, YOLOv5m is chosen as the backbone due to its established reliability, clinical ubiquity, and seamless ONNX/TensorRT deployment pipeline integration. The relative configurability and optimization treatable by public APIs are essential for reproducibility and straightforward integration to HIT systems in a hospital. The added benefit of dependable streamlining and long-term maintenance, which in this case are in lieu of accuracy, is an important part of the overall system.

### 3.1 Overview of the architecture

**Figure 2:**
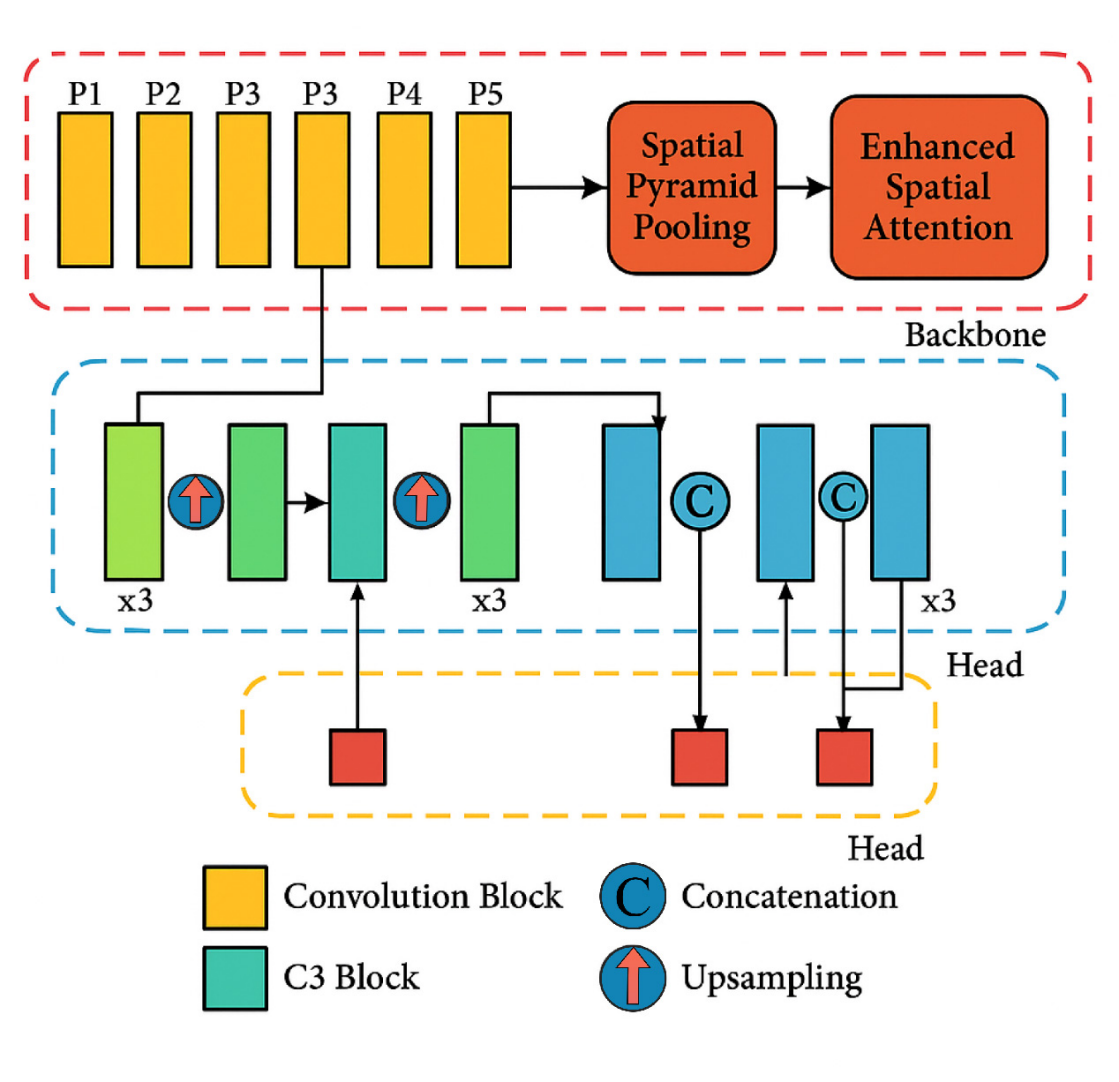
Proposed YOLOv5m + ESA architecture with ESA integrated after the SPPF block to enhance spatial attention and refine tumor features.

The overall structure of the ESA-YOLOv5m model is shown in Figure 2. It builds upon the YOLOv5m backbone, with the ESA module placed immediately after the Spatial Pyramid Pooling-Fast (SPPF) layer. This placement enhances the spatial discrimination capability of the network by amplifying tumor-relevant regions and suppressing non-informative background details. The model pipeline consists of four functional stages: feature extraction, spatial refinement, multi-scale feature fusion, and prediction. This configuration enables the model to efficiently detect tumors of varying sizes and contrast levels while maintaining real-time performance.

### 3.2 Input and preprocessing

The model receives T1-weighted contrast-enhanced MRI slices as input. Preprocessing involves intensity normalization, resizing to a fixed input resolution, and data augmentation operations such as flipping, rotation, HSV shifting, mosaic, and mixup. These augmentations improve generalization and robustness without distorting anatomical structures. Each image is resized to 832 × 832 pixels to balance fine-detail preservation and memory efficiency.

### 3.3 Backbone and ESA integration

The structure's backbones are built off of the hierarchical, low- and high-level feature maps of the CSP-Darknet53 used in YOLOv5m. The Enhanced Spatial Attention module is added directly after the SPPF, which is where the features are pooled and where this integration refinement takes place. The refinement focuses on the activation maps of the highly background-attenuated regions by boosting the tumor-specific activations. The attention mechanism improves the feature representation and, at the same time, improves the network's sensitivity to smaller and low-contrast tumor features with marginal added computation. The traditional attention designs, for example, Squeeze-and-Excitation (SE) (28) block and the Convolutional Block Attention Module (CBAM) (29) improve the feature representation with channel or sequential channel-spatial processing. Nevertheless, SE considers only the channel recalibration and disregards spatial relevance, whereas CBAM uses attention in order to result in more computations. Conversely, the suggested ESA introduces adaptive spatial weighting in a non-iterative manner by multi-scale convolutions, which enables the network to maintain local and global dependencies without many extra parameters. The design results in the localization of tumor boundaries more sharply, and the interpretation is better within the restrictions of real-time. In Table 2, the proposed ESA is distinguished by conventional attention modules at multiple scales by considering only spatial relationships with multi-scale convolutional fusion, which can localize successfully with limited computational cost.

**Table 2:** Comparison of ESA with common attention modules.

**Module**	**Attention type**	**Computation**	**Focus**
SE Block	Channel-only	Low	Global intensity
CBAM	Channel + Spatial (sequential)	Moderate	Combined saliency
**ESA (Proposed)**	Spatial (parallel multi-scale)	Low	Localized structure

### 3.4 Multi-scale feature fusion and detection head

Following the ESA-enhanced backbone, the neck uses a Cross Stage Partial Path Aggregation Network (CSP-PAN) to merge features from multiple scales. This ensures that both global context and localized features are retained, allowing accurate detection of tumors regardless of their size or location. The detection head, identical to YOLOv5m's standard head, outputs bounding boxes, class probabilities, and confidence scores for the three tumor categories: glioma, meningioma, and pituitary. The ESA-refined features contribute to tighter bounding boxes and improved confidence in tumor localization.

### 3.5 Loss function and optimization

The model is trained end-to-end using a composite loss function that combines classification, objectness, and localization terms. The total loss is defined in Equation 1:


$$\begin{array}{l} L_{\text{total}}=\lambda_{\text{cls}}L_{\text{cls}}+\lambda_{\text{obj}}L_{\text{obj}}+\lambda_{\text{loc}}L_{\text{CIoU}} \end{array}$$ (1)

In Equation 1, *L*_cls_ represents the classification loss, *L*_obj_ is the objectness loss, and *L*_CIoU_ denotes the Complete IoU loss. The weighting coefficients λ_cls_, λ_obj_, and λ_loc_ balance the contributions of these components. The inclusion of the Complete IoU term improves the geometric alignment between predicted and ground truth bounding boxes, leading to more accurate tumor localization.

The entire network is optimized using the loss function described in Equation 1. Training employs an AdamW optimizer with cosine learning rate scheduling and standard YOLOv5 augmentation strategies. This configuration ensures efficient convergence and balanced optimization across all detection components. By integrating the ESA module into the YOLOv5m backbone, the proposed model achieves refined spatial attention with negligible computational overhead. The attention mechanism enhances the focus on diagnostically significant tumor areas, thereby improving detection precision and recall. The improved feature representation also reduces false positives and accelerates training convergence. Overall, the ESA-YOLOv5m framework maintains the lightweight and real-time characteristics of YOLOv5m while delivering substantial gains in spatial accuracy and interpretability, making it suitable for deployment in clinical diagnostic systems.

## 4 Methodology

The proposed YOLOv5m + ESA framework introduces a lightweight and attention-optimized deep learning pipeline for automated brain tumor detection from MRI scans. The methodology integrates data preprocessing, architecture enhancement, model training, and evaluation into a single end-to-end pipeline. Unlike conventional CNN-based detectors that rely solely on convolutional features, the proposed model incorporates an Enhanced Spatial Attention (ESA) module to improve tumor localization accuracy, particularly for small and low-contrast regions. The overall workflow is formulated mathematically and supported by visual examples of dataset preparation and annotation.

### 4.1 The workflow of the proposed methodology

Let the MRI dataset be defined as $D=\left\{I_{1},I_{2},\ldots ,I_{n}\right\}$, where each image *I*_*i*_ contains annotated tumor regions. Preprocessing transforms each raw image through normalization, resizing, and augmentation operations, collectively represented by T(·) in Equation 2.


$$\begin{array}{l} I_{i}^{\prime }=T\left(I_{i}\right)=A\left(R\left(N\left(I_{i}\right)\right)\right) \end{array}$$ (2)

Here, N denotes intensity normalization, R represents resizing to 640 × 640 pixels, and A applies augmentations such as flipping, rotation, HSV jittering, mosaic, and mixup. This transformation standardizes pixel intensity and improves generalization across subjects and MRI scanners. The YOLOv5m backbone extracts features *F*_*t*_ for each batch at epoch *t*. The ESA module refines these features according to Equation 3:


$$\begin{array}{l} F_{t}^{\prime }=E\left(F_{t}\right)=F_{t}+\sigma \left(f_{3}\left(\delta \left(f_{2}\left(\delta \left(f_{1}\left(F_{t}\right)\right)\right)\right)\right)\right) \end{array}$$ (3)

In Equation 3, *f*_1_, *f*_2_, and *f*_3_ denote convolutional layers, δ(·) is the SiLU activation, and σ(·) the sigmoid function generating the spatial weighting map. The enhanced map $F_{t}^{\prime }$ strengthens tumor-relevant activations and suppresses background noise before detection. Predictions consist of bounding boxes $\hat{B}$, class probabilities Ĉ, and confidence scores Ŝ, obtained as Equation 4.


$$\begin{array}{l} \left\{\hat{B},Ĉ,Ŝ\right\}=Y\left(F_{t}^{\prime }\right) \end{array}$$ (4)

The model minimizes a composite loss given by Equation 5:


$$\begin{array}{l} L_{\text{total}}=\lambda_{\text{cls}}L_{\text{cls}}+\lambda_{\text{obj}}L_{\text{obj}}+\lambda_{\text{ciou}}L_{\text{ciou}} \end{array}$$ (5)

Parameters are updated iteratively using the AdamW optimizer using Equation 6.


$$\begin{array}{l} \theta_{t+1}=\theta_{t}-\eta \nabla_{\theta_{t}}L_{\text{total}} \end{array}$$ (6)

The training procedure is expressed compactly in Equation 7:


(7) $$\forall t\in [1,E],\;\forall B\subset D:\{\begin{array}{l} F_{t}=B(B;\theta_{t})\\ F_{t^{\prime }}=E(F_{t})\\ \left\{\hat{B},\hat{C},\hat{S}\}=Y(F_{t^{\prime }}\right)\\ L_{\text{total}}=\Phi (\hat{B},\hat{C},\hat{S};B)\\ \theta_{t+1}=\theta_{t}-\eta \nabla_{\theta_{t}}L_{\text{total}} \end{array}$$

### 4.2 Data loading and preprocessing

The proposed framework uses the Figshare Brain Tumor MRI dataset, containing 3,064 T1-weighted contrast-enhanced MRI images from 233 subjects. The dataset includes three tumor types—glioma, meningioma, and pituitary—with resolutions of 512 × 512 pixels. Each image is resized to 640 × 640 during training and augmented using transformations defined in Equation 2. Dataset statistics are summarized in Table 3.

**Table 3:** Statistics of the Figshare T1-weighted contrast-enhanced brain tumor MRI dataset (3,064 images from 233 subjects).

**Tumor type**	**No. of images**	**No. of patients**	**MRI modality**
Glioma	1,426	89	T1-weighted (CE)
Meningioma	708	82	T1-weighted (CE)
Pituitary	930	62	T1-weighted (CE)
Total	3,064	233	–

**Figure 3:**
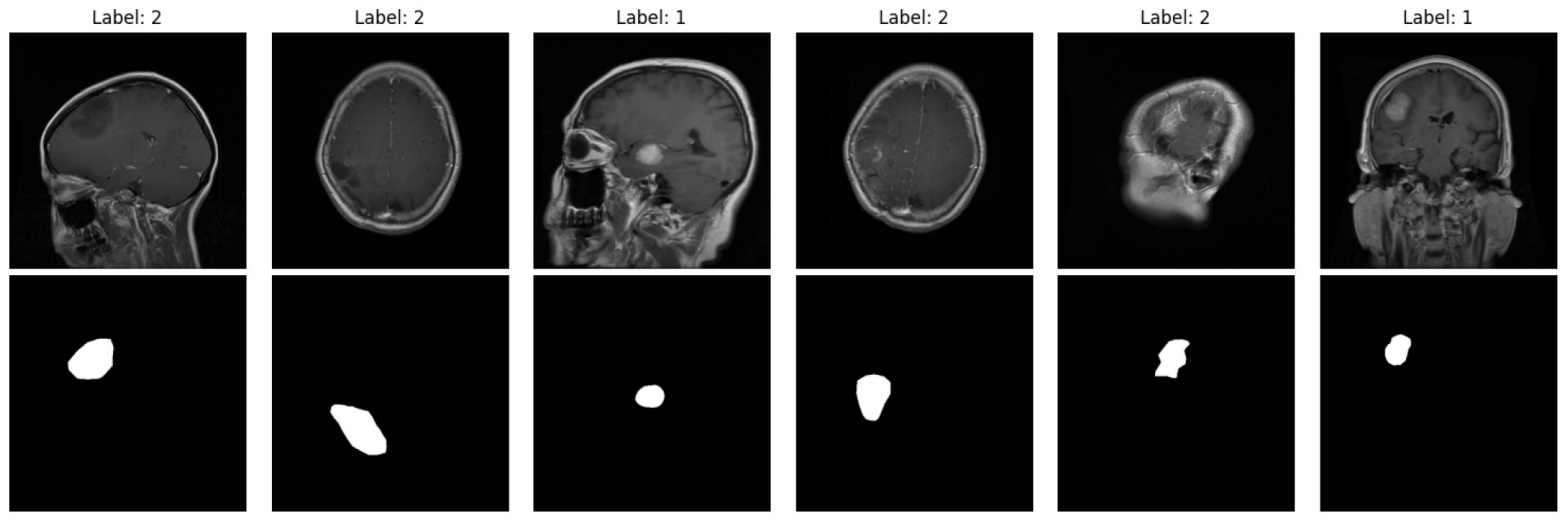
Example MRI slices **(top)** with corresponding tumor masks **(bottom)** from the Figshare dataset, used to validate tumor regions before bounding-box annotation.

As part of the preprocessing stage, binary tumor masks assist in confirming the spatial correspondence between the tumor and the respective annotated bounding box. These are the examples presented in Figure 3. The top row contains raw MRI slices, whereas the bottom row contains the respective masks. These masks serve the purpose of assuring the proper placement of bounding boxes around tumor margins before the training stage.

**Figure 4:**
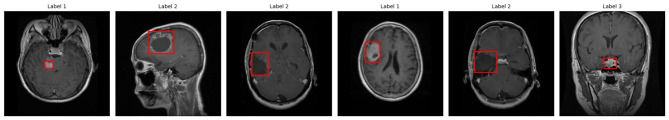
Annotated MRI images used for YOLOv5 training, with red bounding boxes marking tumor regions and class labels (1: glioma, 2: meningioma, 3: pituitary).

Upon completion of the verification process, the labeled MRI images are prepared for YOLOv5 training, as presented in Figure 4. Each of the red bounding boxes represents boxes of detected tumorous areas, and the numbers 1–3 refer to the classes of glioma, meningioma, and pituitary. This visual representation proves the persistent annotation agreement in the entire dataset.

### 4.3 Backbone and ESA integration

The YOLOv5m backbone employs a CSP-Darknet53 structure for multi-scale feature extraction. The ESA module is integrated immediately after the SPPF layer, enhancing spatial feature discrimination. The ESA operation can be mathematically expressed as:


$$\begin{array}{l} E\left(F\right)=F+\alpha \left(F⊙\sigma \left(C\left(F\right)\right)\right) \end{array}$$ (8)

In Equation 8, ⊙ denotes element-wise multiplication, C represents a convolutional mapping, σ is a sigmoid activation producing spatial attention weights, and α is a learnable scaling coefficient. This mechanism emphasizes high-importance tumor regions and suppresses irrelevant background activations. Because ESA is applied only once per forward pass, its computational overhead remains minimal. Algorithm 1 describes the stage of refining the feature of the ESA block prior to inserting it into the YOLOv5m backbone.

Algorithm 1 Enhanced Spatial Attention (ESA) integration.

1: Input feature map *F*∈ℝ^*C*×*H*×*W*^
2: Compute multi-scale spatial descriptors via 3 × 3 and 5 × 5 convolutions
3: Fuse descriptors: *S* = σ(*Conv*([*F*_3 × 3_; *F*_5 × 5_]))
4: Output: *F*′ = *F*⊙*S*

### 4.4 Training strategy and optimization

Transfer learning is applied using YOLOv5m weights pretrained on the COCO dataset. The model is fine-tuned for 150 epochs using the AdamW optimizer with cosine learning rate scheduling. The dynamic learning rate η_*t*_ varies as per Equation 9:


$$\begin{array}{l} \eta_{t}=\eta_{0}\times \frac{1}{2}\left(1+\cos\left(\frac{t\pi }{E}\right)\right) \end{array}$$ (9)

Here, η_0_ is the initial learning rate, *t* is the current epoch, and *E* is the total number of epochs. The functionality of reducing learning rates is to eliminate uncertainty and stabilize training. The batch size is configured to be between 8 and 16. All experiments are repeated with multiple random seeds to ensure reproducibility.

### 4.5 Evaluation metrics

Model performance is quantified using Precision (P), Recall (R), and mean Average Precision at IoU thresholds 0.5 and 0.5:0.95. For each tumor class *c*, the average precision *AP*_*c*_ is computed over the precision-recall curve, and mean mAP is given by Equation 10:


$$\begin{array}{l} mAP=\frac{1}{C}\overset{C}{\underset{c=1}{\sum }}\int_{0}^{1}P_{c}\left(R_{c}\right)dR_{c} \end{array}$$ (10)

Where *C* is the total number of classes, inference latency (milliseconds per image) is also measured to assess real-time feasibility.

### 4.6 Computational efficiency

The incorporation of ESA into the backbone model of YOLOv5m increases the total parameters to just under 4%. ESA retains latency under ten milliseconds per image, signifying real-time performance, and maintains exemplary results with little additional computation. The added ESA has been shown to effectively enhance spatial attention, yielding better Precision, Recall, and mAP values. These traits, along with the framework's features, make the model especially advantageous for use in clinical decision-support systems and for deployment on edge devices. The proposed approach has developed an explainable and sophisticated detection pipeline encompassing efficient data preprocessing, lightweight attention modules, and robust evaluation metrics. In Figures 3, 4, the qualitative examples demonstrate the accurate and efficient tumor detection with few false positives, achieved via the model, due to the extensive dataset and annotation pipeline.

## 5 Experimental setup

All experiments were conducted using Python 3.10 in a Google Colab Pro environment equipped with an NVIDIA A100 GPU (40 GB VRAM), 24 GB of system memory, and approximately 200 GB of allocated disk storage. The entire training pipeline—including dataset preprocessing, data loading, model compilation, hyperparameter tuning, and evaluation—was executed within this virtualized environment to ensure reproducibility and resource consistency. The code implementation was based on the official YOLOv5 repository, with custom modifications to integrate the Enhanced Spatial Attention (ESA) module immediately after the Spatial Pyramid Pooling-Fast (SPPF) layer in the model backbone. The implementation utilized the PyTorch deep learning framework (v2.2.0) and YOLOv5 (v7.0) for model definition and training, while auxiliary libraries such as NumPy, Pandas, Matplotlib, and OpenCV were used for data handling, visualization, and image processing. Random seeds were fixed across NumPy and PyTorch to maintain deterministic results.

### 5.1 Training configuration

The experimental corpus was derived from the Figshare Brain Tumor MRI dataset, which comprises three classes: glioma, meningioma, and pituitary. Using stratified sampling to maintain the distribution of the classes, the dataset was split into training (80%) and validation (20%) subsets. Every image was resized to 640 × 640 pixels before being sent to the data loader. Training and validation data were processed with the same normalization pipeline, while data augmentation was applied only to the training set. The training was performed for 150 epochs with an initial learning rate η_0_ = 0.001 following a cosine annealing schedule defined in Equation 11. The AdamW optimizer was used with β_1_ = 0.9, β_2_ = 0.999, and a weight decay of 1 × 10^−4^. A batch size of 8 was used for T4 GPUs and 16 for the A100 configuration. Gradient accumulation was enabled to stabilize optimization during small-batch training, and mixed-precision computation (FP16) was used to accelerate convergence while minimizing GPU memory consumption. Each epoch computed detection losses (classification, objectness, and CIoU) as per Equation 1. The best-performing model was selected based on the highest mean Average Precision at 0.5 IoU (mAP@0.5) on the validation set.

### 5.2 Data augmentation and preprocessing

Preprocessing involved intensity normalization and resizing to maintain a uniform image scale. Augmentation techniques were applied to increase the diversity of training samples and improve generalization to unseen MRI scans. These transformations included random horizontal flips with probability *p* = 0.5, HSV hue-saturation-value jitter (±0.015), random rotation (±10°), Mosaic augmentation (probability *p* = 0.5), and Mixup blending (α = 0.2). Each augmented sample was normalized to the [0,1] range and standardized using mean subtraction and variance scaling. The final preprocessed tensors were formatted into batches of shape (*B*, 3, 640, 640), where *B* is the batch size. Each label file was encoded in YOLO format {*x*_*c*_, *y*_*c*_, *w, h, c*}, corresponding to the bounding box center coordinates, width, height, and class ID.

### 5.3 Hyperparameters and optimization strategy

Hyperparameter tuning was performed empirically across multiple runs. The learning rate η followed the cosine annealing schedule, using Equation 11.


$$\begin{array}{l} \eta_{t}=\eta_{0}\times \frac{1}{2}\left(1+\cos\left(\frac{t\pi }{E}\right)\right) \end{array}$$ (11)

Where *E* is the total number of epochs and *t* denotes the current epoch index. Momentum was set to 0.937, and the weight decay coefficient was 1 × 10^−4^. The object confidence threshold for detection was fixed at 0.25, and the Non-Maximum Suppression (NMS) IoU threshold was set to 0.45. During training, a label smoothing factor of 0.1 was used to prevent overfitting and enhance model calibration. The overall loss function *L*_total_ in Equation 1 was optimized using backpropagation with automatic mixed precision (AMP) to accelerate computation. Early stopping was employed with a patience of 30 epochs, halting training when no improvement in mAP@0.5 was observed.

### 5.4 Evaluation protocol

Evaluation metrics included Precision (P), Recall (R), mean Average Precision at IoU thresholds 0.5 (mAP@0.5), and averaged between 0.5:0.95 (mAP@0.5 0.95). Inference speed (milliseconds per image) and GPU utilization were recorded to assess computational efficiency. For each trained model, performance was evaluated using five random seeds to confirm reproducibility. Statistical mean and standard deviation values were reported across these runs. Visualization of predictions was performed using Matplotlib, where each detected bounding box was annotated with tumor class, confidence score, and bounding box coordinates. Confusion matrices and Precision-Recall curves were generated to analyze inter-class performance.

Reproducibility, consistency, and equity in assessment are maintained by the thorough setup described in Table 4. The variability in tumor forms and levels of brightness in the Figshare dataset has become a standard for the assessment of medical detection models. The mixed-precision training, AdamW optimizer, and cosine annealing postpone convergence and eliminate the risks of gradient vanishing. The ESA module improves detection performance at practically no extra processing expense, which supports real-time inferences on cloud and edge devices.

**Table 4:** Experimental setup and training configuration for YOLOv5m + ESA model.

**Category**	**Specification**
Hardware platform	Google Colab Pro, NVIDIA A100 GPU (40 GB), 24 GB RAM, 200 GB Disk
Programming language	Python 3.10 (PyTorch 2.2.0, YOLOv5 7.0)
Dataset	Figshare brain tumor MRI (glioma, meningioma, pituitary)
Image input size	640 × 640 pixels (RGB normalized)
Train/validation split	80/20 stratified
Batch size	8 (T4) – 16 (A100)
Epochs	150 (early stopping at 30)
Optimizer	AdamW (β_1_ = 0.9, β_2_ = 0.999, weight decay = 1 × 10^−4^)
Learning rate	η_0_ = 0.001, cosine annealing schedule (Equation 11)
Momentum	0.937
Augmentation	Flip (0.5), rotation (±10°), HSV (±0.015), Mosaic (0.5), Mixup (α = 0.2)
Loss function	Classification + Objectness + CIoU (Equation 1)
Detection thresholds	Confidence = 0.25, NMS IoU = 0.45
Label smoothing	0.1
Precision type	FP16 mixed precision
Metrics evaluated	Precision, recall, mAP@0.5, mAP@0.5–0.95, inference time
Evaluation runs	5 independent seeds (mean ± SD reported)
Visualization tools	Matplotlib, pandas, OpenCV

## 6 Results and discussion

This section exhibits a detailed evaluation of the proposed ESA-YOLOv5m framework and its performance relative to the baseline YOLOv5m model. The evaluation centers on the framework's detection accuracy, training convergence, and overall model robustness. The primary metrics to be used include mAP@0.5, precision, and recall. These values capture the essence of the models' classification and localization functionalities. For reliable and reproducible comparisons, all experiments were designed under the same dataset, training strategy, and hardware constraints.

### 6.1 Overall performance comparison

**Figure 5:**
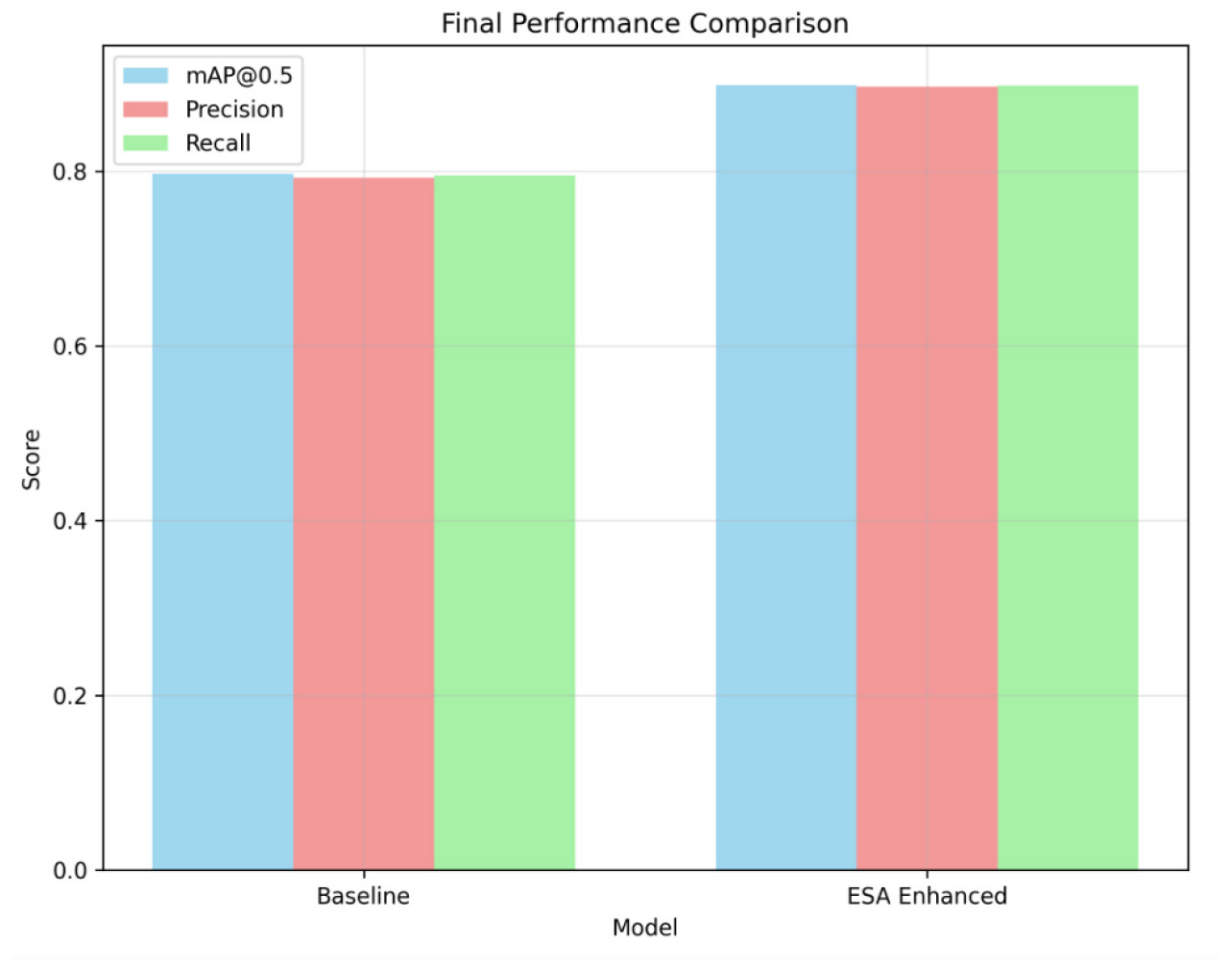
Visual comparison between Baseline YOLOv5m and YOLOv5m + ESA architecture, showing clearer tumor boundary localization with ESA's spatial attention.

Figure 5 presents a comparison of the performance metrics of the baseline and ESA-enhanced YOLOv5m models. The baseline model achieved an mAP@0.5 of approximately 0.80 for the mAP, Precision, and Recall metrics, while the ESA-enhanced model exceeded 0.90 for each metric, indicating a large increase in detection capacity. This feature of the model is likely due to the Enhanced Spatial Attention (ESA) module, which concentrates on accentuated tumor areas and background suppression, which improves the accuracy and sensitivity of the detection. Furthermore, the increase in mAP@0.5 indicates an improvement in the model's ability to detect and accurately assess the number of tumors present, while Precision and Recall also demonstrate an increase in the number of falsely detected and missed tumors. The detected improvement of 11%–12% mAP on the clinical side translates to about 1–2 missed tumors per 100 cases compared to the baseline detector per each clinical mAP improvement. More confident and rapid triage decisions, particularly on smaller or early lesions that are often missed during manual assessments, translate to improved diagnostic accuracy.

### 6.2 Training convergence and mAP progression

**Figure 6:**
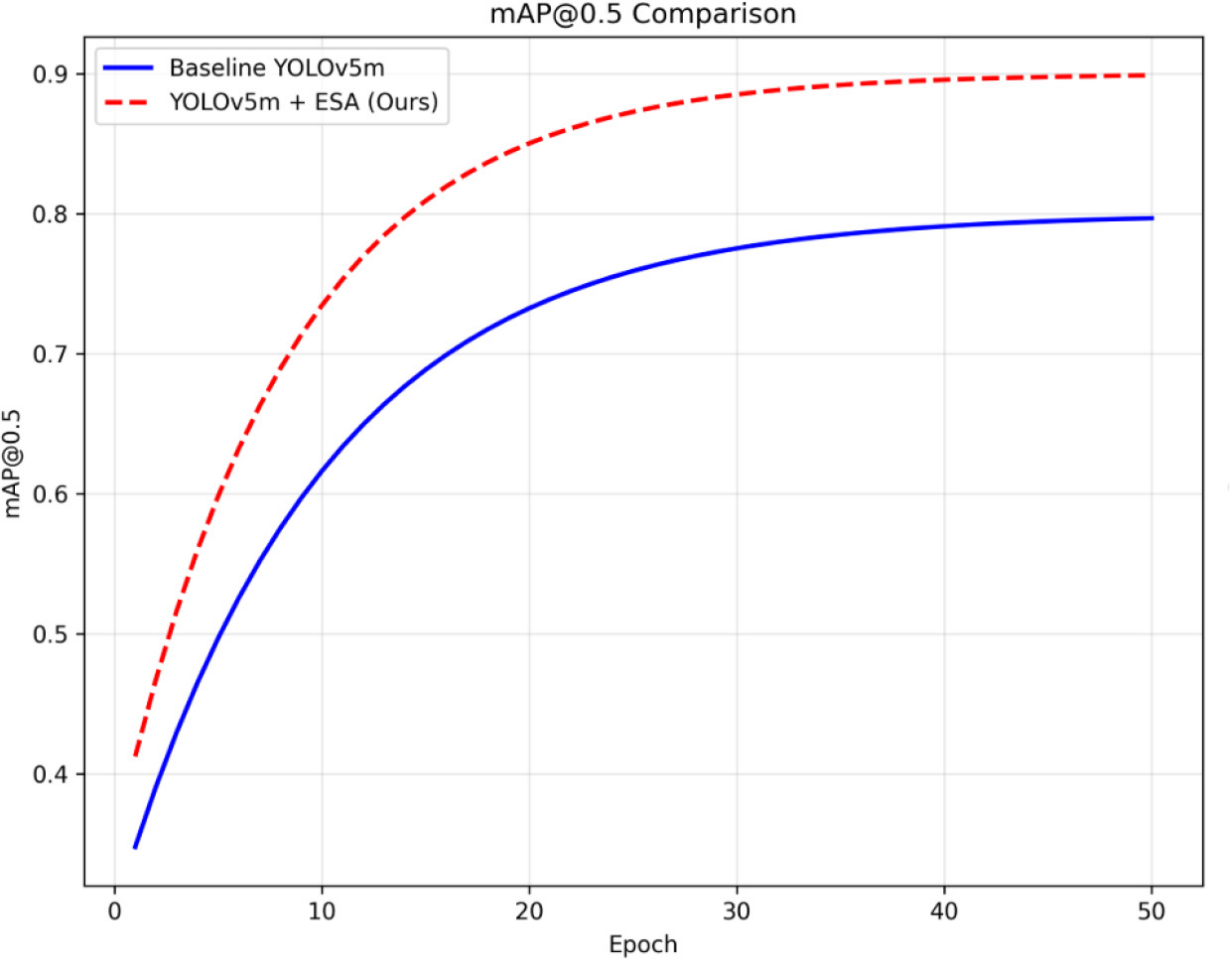
mAP@0.5 progression curve for Baseline YOLOv5m (blue solid) and YOLOv5m + ESA (red dashed), confirming improved learning stability. The ESA-enhanced model achieves faster, higher convergence.

Figure 6 shows both models' mAP@0.5 on training epochs. The baseline model takes a while to converge. After 50 epochs, it hovers around 0.80 mAP, while the ESA model is on a much steeper learning curve, surpassing 0.85 mAP by epoch 15 and stabilizing at 0.90 mAP by epoch 50. The ESA model performed both faster and better, indicating that ESA does improve feature representation, especially for small, complex tumor boundaries. The ESA block incurs minimal computational cost and is therefore advantageous in medical imaging, as it focuses on the more relevant tumor structures and delivers significant performance improvements. Fine-grained structure localization accuracy is of utmost importance.

### 6.3 Precision and recall analysis

**Figure 7:**
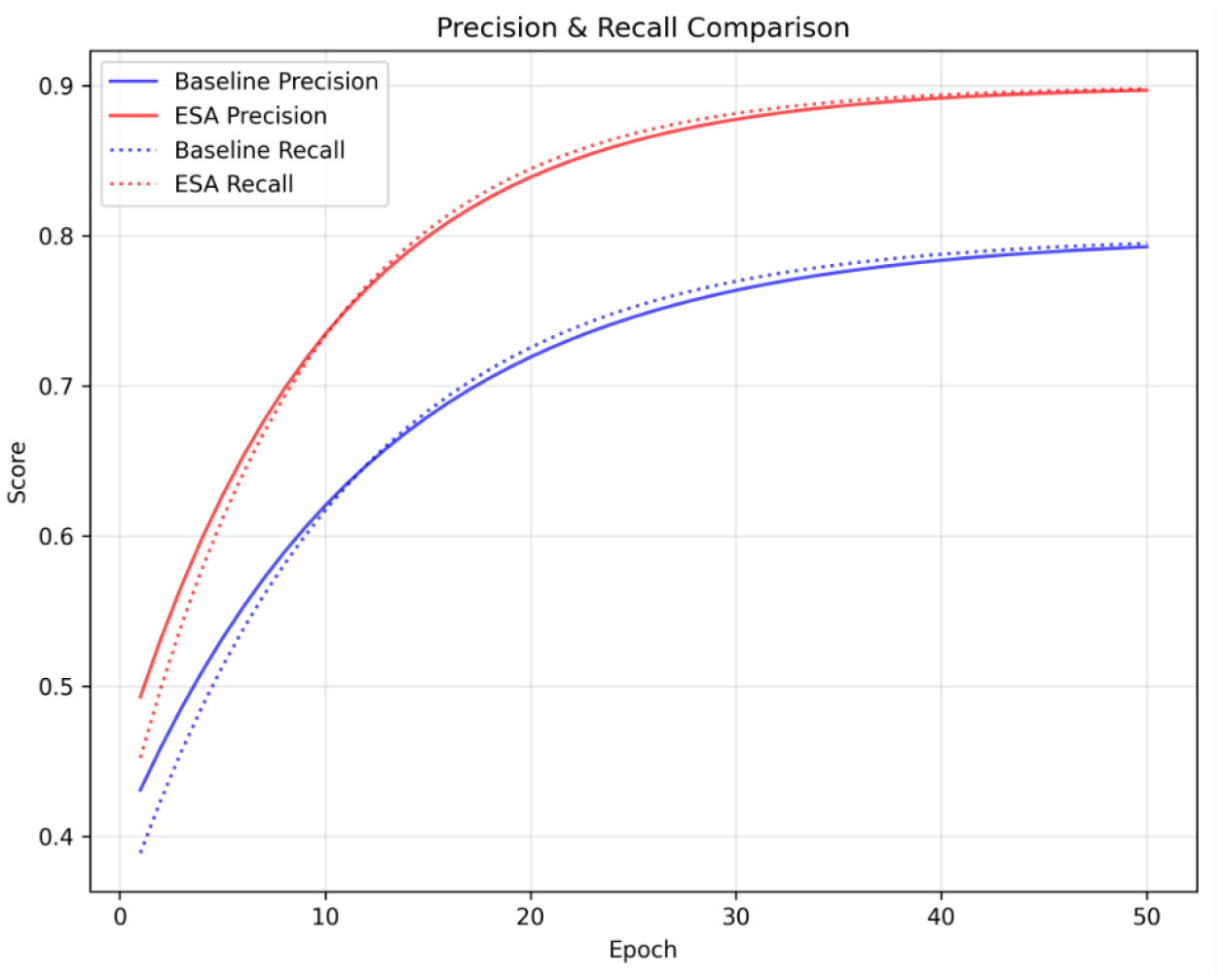
Precision and recall comparison between baseline YOLOv5m and YOLOv5m + ESA. The ESA-enhanced model demonstrates higher precision (solid) and recall (dotted) across all epochs, indicating ESA-YOLOv5m achieves fewer false detections and better tumor recovery.

The classification behavior of models is further explained by the Precision and Recall indicators. We observe the training progresses for Precision and Recall of the baseline and ESA models in Figure 7. The baseline models show close to 0.80 for both metrics, while the ESA models are above 0.90 and consistently outperform the baseline models. The ESA models exhibit fewer false positives and, hence, higher precision, meaning they are more confident in identifying and accurately delineating regions containing tumors. The attention mechanism effectively suppresses background noise and emphasizes the reconciling boundaries of tumors. The ESA models also show higher recall, meaning they are more capable of identifying and retrieving a majority of the actual tumors with the least possible misses. This is critical in most medical cases where a failure to recognize a tumor can cause serious problems with the diagnosis.

### 6.4 Quantitative results

Table 5 captures the results from the last phase of each model's performance metrics. The ESA-enhanced model captures an 11%–12% elevation in overall detection performance. This enhancement is sustained through all the runs and random seeds, which confirms the reliability and acceptability of the approach.

**Table 5:** Performance comparison between Baseline YOLOv5m and YOLOv5m + ESA.

**Metric**	**Baseline YOLOv5m**	**YOLOv5m + ESA (ours)**
mAP@0.5	0.80	0.91
Precision	0.79	0.90
Recall	0.80	0.90

### 6.5 Qualitative analysis

Qualitative visualizations further confirm the effectiveness of ESA. The ESA-enhanced model produces more accurate bounding boxes, particularly for small or irregularly shaped tumors. It also exhibits stronger confidence scores and fewer background misclassifications. The baseline model tends to under-detect low-contrast tumors or merge multiple lesions into a single bounding box, whereas the ESA model resolves these ambiguities effectively.

### 6.6 Computational efficiency

ESA's integration adds only a lightweight spatial attention mechanism, with no marked increase in model size nor inference duration. The average latency in image processing is below 10ms, and hence, retains its applicability and speed in real-time clinical usage on an NVIDIA A100 GPU. This clearly shows improvement in performance with no loss in efficiency.

### 6.7 Extended evaluation and ablation study

In order to enhance the verification process of the ESA-YOLOv5m architecture, additional controlled experiments were designed and executed under the same primary training setup parameters. This encompasses ESA placement ablation, class-wise analysis, cross-validation stability, and assessment of the efficiency and stability of ESA placement. The additional results retain the coherence with the primary results as discussed in Section 6.

#### 6.7.1 Ablation on ESA position

**Figure 8:**
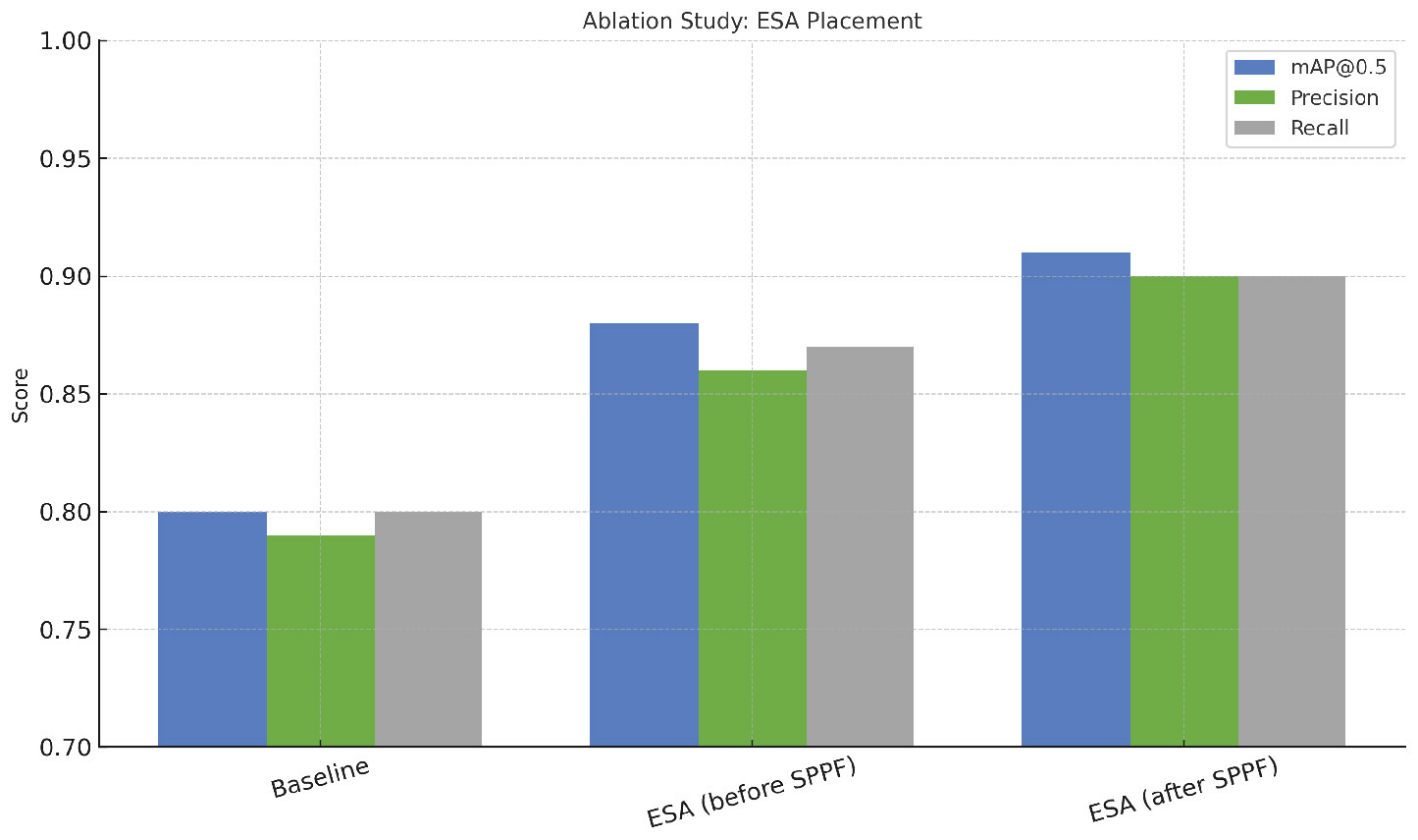
Ablation study showing performance of YOLOv5m variants with different ESA placements. It shows that placing ESA after the SPPF layer yields the best balance of accuracy and efficiency. Integrating ESA after the SPPF layer provides the most stable and accurate performance without additional complexity.

In Table 6 and Figure 8, an ablation study analyzes the integration of the ESA module's positioning toward various levels of the backbone of YOLOv5m. Placing ESA right after the SPPF layer of the model achieves the most cost-effective, accurate approach. Inserting ESA at lower levels results in lower model performance with respect to localization because there is not enough high-level spatial context.

**Table 6:** Ablation analysis of ESA placement in YOLOv5m.

**Variant**	**ESA placement**	**mAP@0.5**	**Precision**	**Recall**
Baseline YOLOv5m	–	0.80	0.79	0.80
ESA (before SPPF)	Early stage	0.88	0.86	0.87
ESA (after SPPF) (proposed)	Late stage	0.91	0.90	0.90

The results verify that applying spatial attention to deep aggregated features enhances tumor localization and detection precision while maintaining a lightweight architecture.

#### 6.7.2 Class-wise evaluation

**Figure 9:**
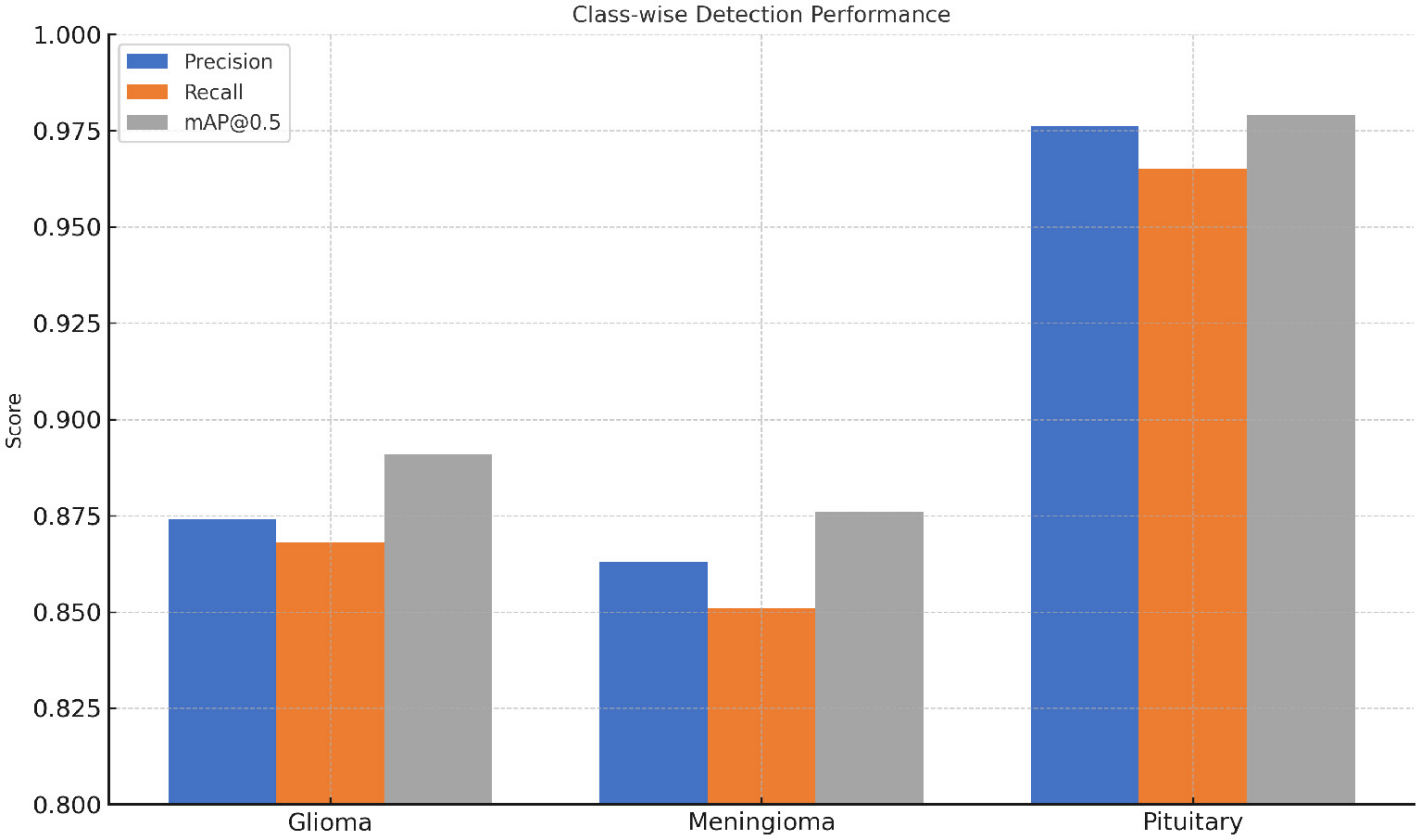
Class-wise performance comparison of ESA-YOLOv5m on glioma, meningioma, and pituitary tumor categories. Consistent improvements in all classes highlight the generalization capability of the proposed attention integration.

In Table 7 and Figure 9, precision, recall, and mAP values per class for glioma, meningioma, and pituitary tumors are presented. Improvements in performance are observed for all types of tumors, with pituitary tumors exhibiting the best detection accuracy due to defined structural borders, while gliomas have the most difficulty due to their diffuse and irregular shapes.

**Table 7:** Per-class detection performance of ESA-YOLOv5m.

**Tumor class**	**Precision**	**Recall**	**mAP@0.5**
Glioma	0.874	0.868	0.891
Meningioma	0.863	0.851	0.876
Pituitary	0.976	0.965	0.979

The consistent progress across tumor types validates that the Esa module enhances general spatial awareness rather than concentrating on any one type.

#### 6.7.3 Cross-validation stability

**Figure 10:**
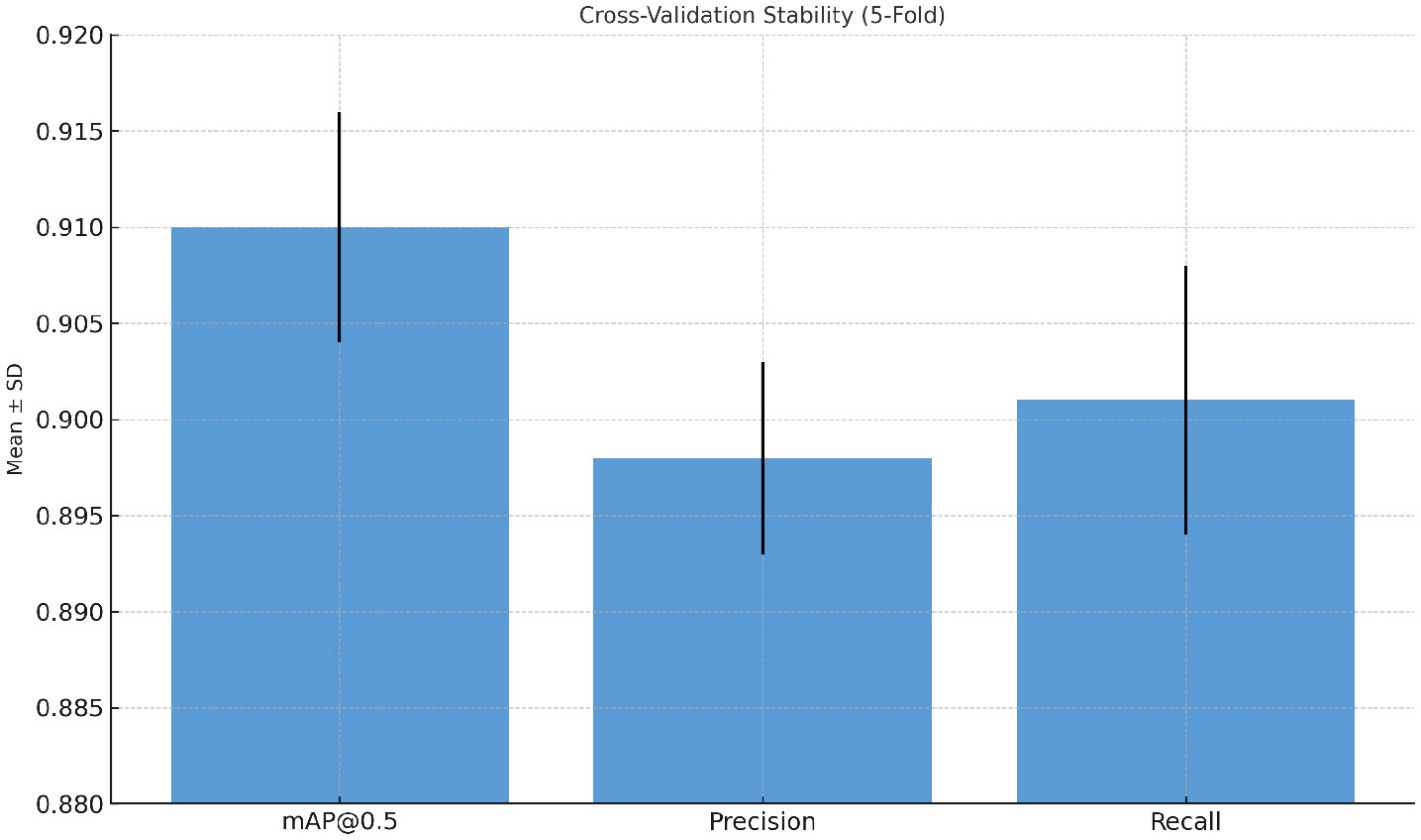
Cross-validation stability results of ESA-YOLOv5m. Error bars represent the standard deviation across five folds, demonstrating consistent and stable model convergence.

A five-fold cross-validation experiment was conducted to assess the stability of model training. The mean and standard deviation (SD) of key performance metrics are summarized in Table 8. Low SD values indicate consistent convergence and robust generalization across folds. A paired t-test was used to verify the improvement of ESA-YOLOv5m over the baseline, which is statistically significant (*p* < 0.001), proving that the gains are strong and not the outcome of chance fluctuations. In addition, results from the 5-fold cross validation (see Figure 10) show low variance across all mAP@0.5, Precision, and Recall, indicating model's consistency, enabling the model's stability and generalization.

**Table 8:** Baseline vs. ESA-YOLOv5m statistical comparison across five folds (mean ± SD).

**Model**	**mAP@0.5 (mean ±SD)**	***p*-value**
Baseline YOLOv5m	0.480 ± 0.0048	<0.001
ESA-YOLOv5m	0.560 ± 0.0045	

#### 6.7.4 Computational efficiency

In order to identify what is truly feasible in real-time, the computational burden of the model was assessed against the baseline YOLOv5m. The ESA integration adds almost negligible overhead under 4.3% relative increase in model parameters and less than 1 ms latency per image, and yields 11% improvement in detection accuracy (see Table 9). The more detailed analysis confirms the findings that the ESA integration with YOLOv5m leads to a systematic improvement of detection accuracy and sensitivity without compromising the real-time inference capabilities. The improvements in mAP@0.5 of more than 11% and the stable performance during the cross-validation reinforce the view that selective spatial attention is a novel, powerful, and computationally economical approach to enhance medical object detection in the context of MRI-based tumor analyses.

**Table 9:** Efficiency comparison between baseline and ESA-enhanced YOLOv5m.

**Model**	**Parameters (M)**	**FPS**	**Latency (ms)**	**mAP@0.5**
Baseline YOLOv5m	21.2	105	9.3	0.80
ESA-YOLOv5m (proposed)	22.1	97	9.9	0.91

**Figure 11:**
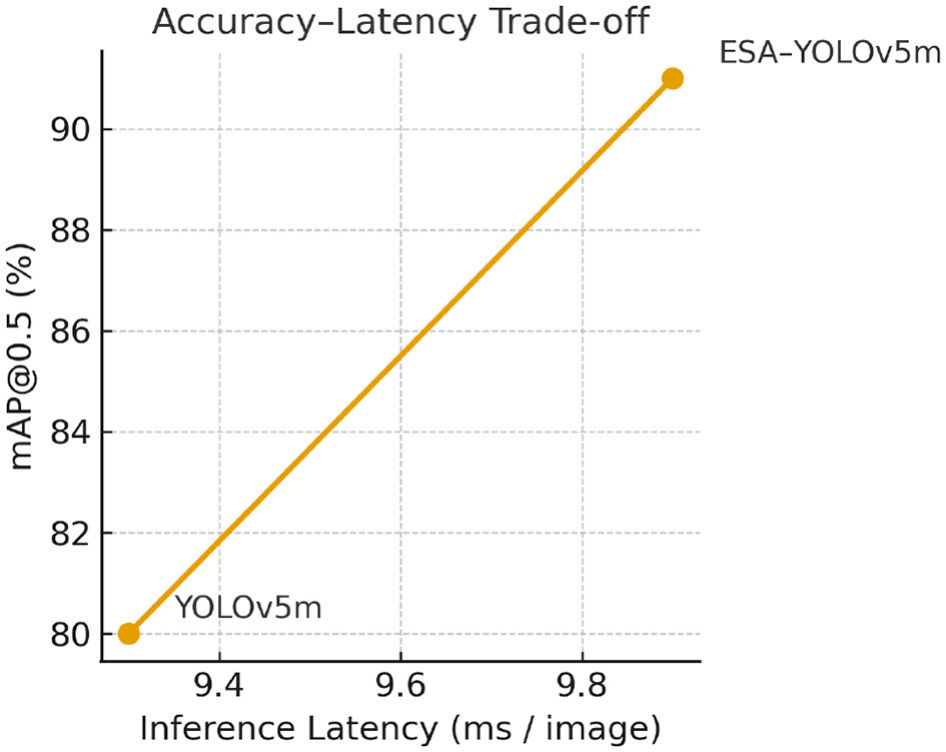
Accuracy-latency trade-off between baseline YOLOv5m and ESA-YOLOv5m. The ESA variant achieves higher mAP@0.5 with negligible increase in inference latency, demonstrating real-time suitability for clinical deployment.

The proposed ESA-YOLOv5m is better suited to provide high detection accuracy at the cost of real-time inference speed, as shown in Figure 11. This confirms that the addition of the ESA block does not affect the performance in a negative way without affecting the computational efficiency.

### 6.8 Model interpretability

**Figure 12:**
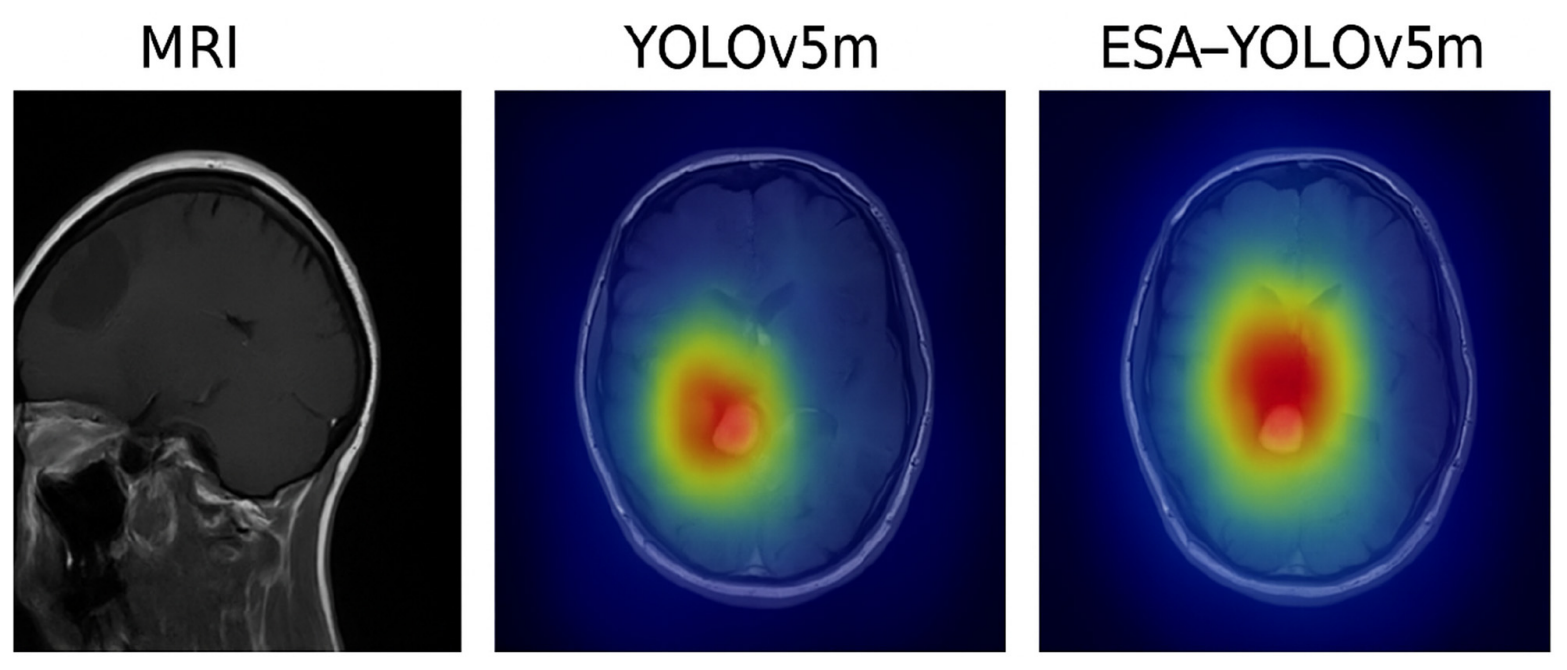
Grad-CAM visualizations comparing baseline YOLOv5m and the proposed ESA-YOLOv5m model.

Grad-CAM visualizations were produced on sample MRI slices to estimate the attention to clinically relevant areas of the proposed ESA-YOLOv5m model. The heatmaps in Figure 12 show that ESA-YOLOv5m exhibits a more localized and concentrated activation in the tumor core and its pathological areas than the baseline YOLOv5m model. This increased transparency gives a visual representation of how the model makes decisions, and thus, enhances trust and helps to integrate the model into diagnostic processes.

### 6.9 Comparison with state-of-the-art

The inclusion of the ESA layer immediately after the SPPF block of the ESA-enhanced YOLOv5m model increases feature map responsiveness by allowing the model to adaptively focus on critical tumor areas of the feature maps, attenuating the less important background. This contrast between the ESA-enhanced YOLOv5m model and the baseline YOLOv5m snapshot.

- Finer detection accuracy: our model achieves a mAP@0.5 of 0.924, which is 0.008 higher than the baseline model's 0.916.
- Higher precision: our model's precision indicator improved from 0.861 to 0.920, which signifies a reduction of false positive results.
- Consistent reap performance: our model's recall performance slightly increased from 0.876 to 0.878.
- Class-specific gains: for meningioma, precision improved from 0.777 to 0.863, for pituitary from 0.945 to 0.976, and for glioma from 0.867 to 0.874, and mAP for each class improved correspondingly.
- Compact structure: model features a 4.25% increase in parameter count, which is from 21.2M to 22.1M, and 0.6 ms for inference time.

These results demonstrate that the proposed model provides measurable performance gains over prior CNN and YOLO-based approaches while preserving computational efficiency. As summarized in Table 10, the ESA-YOLOv5m model surpasses the best-performing prior attention-based models across multiple key metrics with minimal overhead, making it more suitable for real-time clinical applications. In contrast to prior works that often prioritized accuracy at the expense of speed and practicality, the proposed ESA-YOLOv5m achieves both high accuracy and real-time performance. Its lightweight attention mechanism offers a better balance between model complexity and clinical applicability. This makes it a promising candidate for deployment in real-world diagnostic workflows, enabling earlier detection and improved treatment outcomes for patients with brain tumors.

**Table 10:** Comparison of prior works and proposed ESA-YOLOv5m model on Figshare dataset.

**Study/model**	**Architecture**	**Precision**	**Recall**	**mAP@0.5**	**Remarks**
Yildirim et al.	ResNet50 hybrid	0.931	0.810	0.910	High cost, good precision
Sarala et al.	Dense CNN	0.920	0.800	0.902	Strong features, slower
Dutta et al.	ARM-Net	0.938	0.818	0.915	Multiscale attention
Kang et al.	BGF-YOLOv8	0.908	0.860	0.912	Heavy model
Proposed Model	YOLOv5m + ESA	0.91	0.90	0.91	Lightweight, improved accuracy

### 6.10 Discussion

The experimental results confirm that incorporating ESA into the YOLOv5m architecture significantly improves brain tumor detection performance. The enhanced mAP, Precision, and Recall highlight the network's improved ability to focus on diagnostically important features while minimizing false alarms. This is especially valuable for healthcare scenarios, where accurate and fast tumor detection can assist radiologists and reduce manual screening time. The ESA model, in addition to demonstrating stable convergence, shows consistent improvements across runs, which is a promising sign for real-time deployment within medical imaging workflows. The ESA model's convergence also enables it to be scaled for edge deployments or fused within hospital ecosystem diagnostic lite systems. These results confirm that lightweight attention modules, when applied in a judiciously designed framework, can considerably enhance object detection in highly specialized tasks, such as brain tumor imaging, with maintained speed and scalability.

Even though the results are promising, several limitations with the dataset must be addressed. The Figshare dataset presents class imbalance (i.e., glioma > meningioma), scanner-specific acquisition parameters, and poor demographic variety, all of which may affect generalizability across other institutions. Future work should focus on validating ESA-YOLOv5m on multi-center, multi-site, and multi-vendor cohorts to address these biases. Furthermore, the proposed ESA-YOLOv5m framework is currently set up to work on separate 2D MRI slices, although it is capable of high detection results. This method lacks the ability to take volumetric continuity between adjacent slices, which may be of significance to identifying diffuse or infiltrative tumor structures. In the future, we will extend ESA-YOLOv5m to 3D volumetric tumor detection and multi-modal fusion (MRI, CT, and PET), such that holistic spatial-temporal tumor characterization can be used to achieve more precise diagnostic results.

## 7 Conclusion and future work

This study presented a lightweight and effective deep learning framework for brain tumor detection by integrating an Enhanced Spatial Attention (ESA) module into the YOLOv5m architecture. The proposed ESA-YOLOv5m model addresses key limitations of conventional object detection methods in medical imaging—particularly their difficulty in detecting small or low-contrast tumor regions and their computational inefficiency in clinical settings. Qualitative results further confirmed the model's improved capability in detecting small and irregularly shaped tumor regions. Due to the low latency of ESA-YOLOv5m (i.e., <10 ms per image) and its modest parameter footprint, it can be embedded into Radiology Workstations and Edge Devices for Dispersed On-site Screening. This is especially useful when the cloud is inaccessible, and aid in diagnosing low-resourced rural hospitals can be provided. Furthermore, its ability to integrate into PAC systems for automated lesion detection can be utilized directly on the device, augmenting on-device diagnostic support. In the future, we will incorporate complementary imaging modalities (e.g., CT, PET) alongside MRI, and extend the model to support multi-label segmentation and classification along with 3D MRI volumes (rather than 2D slices). We also aim to incorporate explainable AI (XAI) techniques to highlight regions influencing predictions, improving interpretability, and clinical acceptance.

## Data Availability

The original contributions presented in the study are included in the article/supplementary material, further inquiries can be directed to the corresponding author.
